# Supplementary material for: Preharvest Mandarin Rind Disorder: Insights into Varietal Differences and Preharvest Treatments Effects on Postharvest Quality
Source: Plants (Basel). 2024 Apr 9;13(8):1040. doi: 10.3390/plants13081040 (PMC11053722; doi:10.3390/plants13081040)
Supplement: Supplementary file 1 [file plants-13-01040-s001.zip › plants-2905911-supplementary.pdf]

Supplementary Data

Table S-1: Fruit color index of four mandarin varieties during postharvest in response to various treatments in 2019 (A) and 2020 (B) seasons. Fruits were stored at 0.5 °C and 7.5 °C for four weeks and then for one week at 20 ° C. Data are average of four replicates, and each contains 20 fruit, ± the standard error, letters indicate the statistical difference among treatments and varieties at the level of 0.05.

A

	0.5 °C					7.5 °C					
	Control	Vapor Gard	2,4-D	GA	Mean	Control	Vapor Gard	2,4-D	GA	Mean	
Owari	0.47±0.002a	0.46±0.004a	0.47±0.01a	0.14±0.06b	0.38±0.04b	0.52±0.01a	0.52±0.002a	0.50±0.004a	0.38±0.04b	0.48±0.02c	0.43±0.02B
Page	0.50±0.01a	0.49±0.01ab	0.46±0.01a	0.34±0.02b	0.45±0.02a	0.63±0.01a	0.61±0.01a	0.60±0.01a	0.54±0.03b	0.60±0.01a	0.52±0.02A
W. Murcott	0.56±0.01a	0.52±0.01a	0.54±0.01a	0.31±0.04b	0.48±0.03a	0.59±0.01a	0.55±0.01a	0.55±0.02a	0.41±0.02b	0.52±0.02b	0.50±0.02A
Tango	0.50±0.01a	0.48±0.01ab	0.40±0.02b	0.22±0.07c	0.38±0.04b	0.52±0.01a	0.51±0a	0.50±0.02a	0.29±0.05b	0.44±0.04d	0.44±0.02B
	0.51±0.01a	0.49±0.01ab	0.46±0.01b	0.24±0.03c		0.57±0.01a	0.54±0.01a	0.54±0.01a	0.39±0.04b		
	0.43±0.02A					0.52±0.01B					

B

	0.5 °C					7.5 °C					
	Control	Vapor Gard	2,4-D	GA	Mean	Control	Vapor Gard	2,4-D	GA		
Owari	0.46±0.01a	0.47±0.01a	0.45±0.01a	0.3±0.01b	0.42±0.02d	0.49±0.01a	0.48±0.01a	0.49±0.01a	0.38±0.02b	0.46±0.01c	0.43±0.01C
Page	0.55±0.01a	0.52±0.004a	0.52±0.01a	0.37±0.02b	0.49±0.02c	0.64±0.01a	0.65±0.01a	0.61±0.003b	0.52±0.01c	0.60±0.01a	0.52±0.01A
W. Murcott	0.58±0.01a	0.52±0.01b	0.54±0.01ab	0.40±0.04c	0.51±0.02b	0.58±0.01a	0.54±0.01a	0.56±0.003a	0.46±0.01b	0.54±0.01b	0.5±0.01B
Tango	0.6±0.01a	0.52±0.01a	0.48±0.01a	0.44±0.01a	0.53±0.01a	0.48±0.003a	0.52±0.01b	0.6±0.01a	0.44±0.01b	0.52±0.01b	0.44±0.004B
	0.54±0.01a	0.50±0.01b	0.52±0.01b	0.40±0.02c		0.56±0.01a	0.55±0.02b	0.55±0.01b	0.46±0.01c		
	0.49±0.01B					0.53±0.01A					

Table S-2: Fruit sugar content (Brix) of four mandarin varieties during postharvest in response to various treatments in 2019 (A) and 2020 (B) seasons. Fruits were stored at 0.5 °C and 7.5 °C for four weeks and then for one week at 20 ° C. Data are average of four replicates, and each contains 20 fruit, ± the standard error, letters indicate the statistical difference among treatments and varieties at the level of 0.05.

A

	0.5 °C					7.5 °C					
	Control	Vapor Gard	2,4-D	GA	Mean	Control	Vapor Gard	2,4-D	GA	Mean	
Owari	13.85±0.16a	13.35±0.35ab	13.7±0.16a	12.53±0.5b	13.36±0.20a	13.7±0.2a	13.35±0.09a	13.4±0.24a	11.88±0.17b	13.08±0.20a	13.22±0.14a
Page	12.03±0.47a	12±0.48a	11.68±0.21a	11.43±0.48a	11.78±0.20c	11.88±0.25a	12.05±0.51a	11.8±0.25a	11.8±0.31a	11.88±0.16b	11.83±0.13c
W. Murcott	13.13±0.29a	12.43±0.5ab	12.43±0.13ab	11.98±0.39b	12.49±0.19b	13.38±0.52a	12.6±0.16ab	12.93±0.37a	11.8±0.27b	12.68±0.22a	12.58±0.14b
Tango	11.95±0.28a	11.18±0.20ab	11.15±0.18ab	10.73±0.36b	11.25±0.16d	12.775±0.15a	12.15±0.25ab	12.03±0.3ab	11.5±0.29b	12.11±0.17b	11.68±0.14c
	12.74±0.25a	12.24±0.27b	12.24±0.26b	11.66±0.26c		12.93±0.23a	12.54±0.19a	12.54±0.22a	11.74±0.12b		
	12.22±0.14a					12.44±0.11a					

B

	0.5 °C					7.5 °C					
	Control	Vapor Gard	2,4-D	GA	Mean	Control	Vapor Gard	2,4-D	GA		
Owari	12.05±2.83a	12.35±0.16a	12.68±0.23a	8.48±0.16b	11.39±0.77b	11.73±0.17b	11.68±0.14b	12.28±0.05a	10.45±0.18c	11.53±0.18b	11.46±0.39b
Page	12.63±0.34a	12.35±0.12a	12.2±0.2a	11.3±0.25b	12.12±0.17ab	13.48±0.08a	12.78±0.18ab	12.18±0.33b	12.2±0.34b	12.66±0.18a	12.39±0.13a
W. Murcott	13.13±0.29a	12.43±0.5ab	11.98±0.39b	12.43±0.13ab	12.49±0.19a	13.38±0.52a	12.6±0.16ab	11.8±0.27b	12.93±0.37a	12.68±0.22a	12.58±0.14a
Tango	10.23±0.26a	9.63±0.3ab	9.18±0.23b	10.1±0.46ab	9.78±0.18c	9.45±0.22b	9.45±0.54b	9.45±0.25b	10.775±0.5a	9.78±0.23c	9.78±0.14c
	12.01±0.31a	11.69±0.33a	11.51±0.37ab	10.58±0.75b		12.01±0.44a	11.63±0.37ab	11.43±0.32b	11.59±0.3ab		
	11.44±0.24a					11.66±0.18a					

Table S-3: Fruit titratable acidity of four mandarin varieties during postharvest in response to various treatments in 2019 (A) and 2020 (B) seasons. Fruits were stored at 0.5 °C and 7.5 °C for four weeks and then for one week at 20 ° C. Data are average of four replicates, and each contains 20 fruit, ± the standard error, letters indicate the statistical difference among treatments and varieties at the level of 0.05.

A

	0.5 °C					7.5 °C					
	Control	Vapor Gard	2,4-D	GA	Mean	Control	Vapor Gard	2,4-D	GA	Mean	
Owari	1.64775±0.0ab	1.67475±0.1ab	1.7665±0.09a	1.499±0.07b	0.94±0.02c	1.68±0.06a	1.5±0.02b	1.57±0.04ab	1.36±0.04c	1.53±0.03b	1.59±0.028B
Page	0.09±0.05a	0.45±0.22a	0.83±0.41a	0.09±0.04a	1.27±0.01a	1.23±0.03a	1.26±0.06a	1.2±0.06a	1.23±0.07a	1.23±0.03d	1.36±0.062C
W. Murcott	1.67±0.06a	1.57±0.05ab	1.59±0.05a	1.4±0.06b	1.13±0.03b	1.55±0.06a	1.41±0.03ab	1.47±0.08ab	1.3±0.03b	1.43±0.03c	1.49±0.027B
Tango	1.81±0.13a	1.66±0.045ab	1.68±0.06ab	1.50±0.087b	0.84±0.02d	1.86±0.06a	1.78±0.05ab	1.88±0.11a	1.56±0.08b	1.77±0.05a	1.71±0.035A
	1.09±0.04a	1.069±0.03a	1.06±0.03a	0.979±0.04b		1.59±0.06a	1.48±0.06b	1.54±0.07ab	1.35±0.04c		
0.43±0.033A						0.52±0.03B					

B

0.5 °C						7.5 °C					
	Control	Vapor Gard	2,4-D	GA	Mean	Control	Vapor Gard	2,4-D	GA	Mean	
Owari	0.99±0.07a	1.07±0.05a	1.04±0.07a	0.98±0.03a	1.02±0.03c	0.9±0.02ab	0.93±0.06a	0.8±0.04bc	0.77±0.02c	0.85±0.03c	0.94±0.02C
Page	1.33±0.01a	1.25±0.04a	1.29±0.03a	1.33±0.07a	1.30±0.02a	1.33±0.03a	1.22±0.03b	1.2±0.04b	1.25±0.02ab	1.25±0.02a	1.27±0.02A
W. Murcott	1.24±0.09a	1.18±0.09a	1.2±0.06a	0.89±0.03b	1.13±0.05b	1.24±0.03a	1.11±0.05b	1.18±0.02ab	1.02±0.01c	1.14±0.02b	1.13±0.07B
Tango	0.93±0.05a	0.89±0.07a	0.87±0.02a	0.9±0.06a	0.89±0.02d	0.75±0.02bc	0.86±0.06ab	0.88±0.04a	0.67±0.01c	0.79±0.03d	0.84±0.02D
	1.12±0.05a	1.10±0.05ab	1.10±0.05ab	1.02±0.05b		1.05±0.06a	1.03±0.04a	1.01±0.05a	0.93±0.06b		
	1.09±0.02A					1.01±0.03B					

Supplementary Table 4: Fruit PH of four mandarin varieties during postharvest in response to various treatments in 2019 (A) and 2020 (B) seasons. Fruits were stored at 0.5 °C and 7.5 °C for four weeks and then for one week at 20 ° C. Data are average of four replicates, and each contains 20 fruit, ± the standard error, letters indicate the statistical difference among treatments and varieties at the level of 0.05.

A

	0.5 °C					7.5 °C					
	Control	Vapor Gard	2,4-D	GA	Mean	Control	Vapor Gard	2,4-D	GA	Mean	
Owari	3.47±0.05a	3.43±0.06a	3.45±0.05a	3.52±0.09a	3.47±0.07c	3.46±0.05c	3.47±0.04bc	3.53±0.02ab	3.56±0.06a	3.51±0.06d	3.49±0.06d
Page	3.52±0.05a	3.5±0.11a	3.58±0.11a	3.57±0.06a	3.54±0.08b	3.6±0.08a	3.58±0.07a	3.63±0.05a	3.62±0.05a	3.61±0.06c	3.58±0.08c
W. Murcott	3.69±0.09ab	3.66±0.06b	3.66±0.04b	3.77±0.07a	3.7±0.07a	3.79±0.08ab	3.77±0.02b	3.81±0.09ab	3.87±0.05a	3.81±0.07a	3.75±0.090a
Tango	3.61±0.13a	3.64±0.06a	3.64±0.06a	3.73±0.09a	3.66±0.09a	3.62±0.05b	3.65±0.04b	3.7±0.04ab	3.76±0.11a	3.68±0.08b	3.67±0.087b
	3.57±0.12b	3.56±0.11b	3.58±0.12b	3.65±0.13a		3.62±0.13b	3.63±0.1b	3.65±0.14b	3.7±0.14a		
3.59±0.12b						3.65±0.13a					

B

	0.5 °C					7.5 °C					
	Control	Vapor Gard	2,4-D	GA	Mean	Control	Vapor Gard	2,4-D	GA	Mean	
Owari	3.80±0.06a	3.71±0.09a	3.78±0.05a	3.78±0.06a	3.77±0.07b	3.92±0.03a	3.95±0.08a	3.92±0.10a	3.97±0.04a	3.94±0.07a	3.85±0.11b
Page	3.42±0.05a	3.48±0.05a	3.48±0.01a	3.46±0.04a	3.46±0.04d	3.53±0.06a	3.55±0.02a	3.58±0.02a	3.55±0.04a	3.55±0.04c	3.51±0.06d
W. Murcott	3.61±0.05b	3.62±0.09b	3.61±0.01b	3.8±0.01a	3.66±0.09c	3.71±0.04bc	3.77±0.06b	3.69±0.02c	3.86±0.03a	3.76±0.08b	3.70±0.099c
Tango	3.85±0.08a	3.91±0.05a	3.86±0.03a	3.87±0.06a	3.87±0.06a	3.93±0.03b	3.92±0.05b	3.93±0.04b	4±0.04a	3.95±0.05a	3.91±0.064a
	3.67±0.18b	3.7±0.18ab	3.66±0.15b	3.73±0.17a		3.77±0.18b	3.79±0.17b	3.79±0.17b	3.85±0.19a		
3.69±0.17b						3.80±0.17a					

Table S-5: Fruit TSS/Acid ratio of four mandarin varieties during postharvest in response to various treatments in 2019 (A) and 2020 (B) seasons. Fruits were stored at 0.5 °C and 7.5 °C for four weeks and then for one week at 20 ° C. Data are average of four replicates, and each contains 20 fruit, ± the standard error, letters indicate the statistical difference among treatments and varieties at the level of 0.05.

A

	0.5 °C					7.5 °C					
	Control	Vapor Gard	2,4-D	GA	Mean	Control	Vapor Gard	2,4-D	GA	Mean	
Owari	8.42±0.32a	7.81±0.70a	7.99±0.30a	8.37±0.46a	8.15±0.5a	8.17±0.42b	8.56±0.39ab	8.88±0.30a	8.72±0.47ab	8.58±0.44b	8.36±0.52b
Page	8.69±0.47a	7.77±1.75a	9.07±0.87a	8.07±3.12a	8.4±1.74a	9.48±0.79a	9.84±0.60a	9.97±1.33a	9.63±0.73a	9.73±0.83a	9.06±1.50a
W. Murcott	7.86±0.46b	7.94±0.32b	7.86±0.37b	8.55±0.42a	8.05±0.47a	8.68±0.88a	8.93±0.43a	8.85±0.69a	9.08±0.34a	8.88±0.58b	8.47±0.66b
Tango	6.69±0.79a	6.75±0.20a	6.67±0.55a	7.21±0.58a	6.83±0.56a	6.88±0.42ab	6.84±0.21ab	6.46±0.63b	7.43±0.44a	6.90±0.54c	6.87±0.54c
	7.91±0.92a	7.61±0.96a	7.85±1.05a	8.05±1.54a		8.30±1.14a	8.62±1.19a	8.46±1.51a	8.71±0.96a		
	7.86±1.13b					8.53±1.20a					

B

	0.5 °C					7.5 °C					
	Control	Vapor Gard	2,4-D	GA	Mean	Control	Vapor Gard	2,4-D	GA	Mean	
Owari	12.30±1.36a	12.29±1.72a	11.60±1.11a	11.29±0.0.55a	11.91±1.24a	13±0.38b	15.57±1.75a	12.73±1.60b	13.56±0.93b	13.71±1.63a	12.84±1.70a
Page	9.52±0.47a	9.92±0.62a	9.46±0.19a	8.52±0.52b	9.36±0.68c	10.14±0.57ab	10.5±0.54a	10.15±0.20ab	9.79±0.24b	10.15±0.46d	9.75±0.70c
W. Murcott	10.73±1.71b	10.78±2.14b	10.13±1.66b	14.09±1.17a	11.43±2.21ab	10.85±1.07bc	11.45±1.22ab	9.97±0.62c	12.65±0.72a	11.23±2c	11.33±1.79b
Tango	11.08±0.76a	11.01±1.13a	10.55±0.68a	11.32±0.78a	10.99±0.82b	12.68±0.74b	11.02±0.99bc	10.83±0.68c	16.22±1.68a	12.68±1.31b	11.84±1.99b
	10.91±1.47a	10.83±1.37a	10.61±1.55a	11.3±2.23a		11.67±1.40b	11.42±1.34b	11.63±2.53b	13.06±2.55a		
	10.91±1.66b					11.94±2.10a					

Table S-6: Total monthly precipitation from color break stage to harvest during the two years of the study. Data obtained from CIMS https://cimis.water.ca.gov/

	Total Precipitation (mm)	
	2019/2020	2020/2021
**October**	0	0
**November**	26	10.2
**December**	57.8	24.2
**January**	14.8	50.9
**February**	6.9	18.7
**March**	51.7	29.7

Table S-7: Fruit quality of the four mandarin varieties as affected by years. Data are average of four replicates, and each contains 20 fruit, ± the standard error, letters indicate the statistical difference between years at the level of 0.05.

	**Varieties**			
	**Satsuma**	**Page**	**W. Marcotte**	**Tango**
**Fruit weight (g)**				
2019	92.42±1.40**b**	102.18±3.99**a**	79.02±4.67**a**	102.25±3.57**a**
2020	130.25±27.22**a**	86.50±3.63**b**	83.13 ±10.19**a**	65.88±2.13**b**
**Fruit width (cm)**				
2019	5.83±0.05**a**	5.54±0.14**a**	7.20±0.30**a**	4.38±0.15**b**
2020	5.71±0.15**a**	5.28±0.06**b**	4.62±0.27**b**	6.567±0.22**a**
**Fruit length (cm)**				
2029	6.16±0.05**b**	5.90±0.04**a**	8.69±0.25**a**	5.53 ±0.18**b**
2020	7.13±0.19**a**	5.55±0.13**b**	5.85±0.22**b**	8.29±0.27**a**
**Fruit Color Index (a/b**)				
2029	0.38±0.03**a**	0.58±0.02**a**	0.50 ±0.57**a**	0.49±0.54**a**
2020	0.31±0.05**b**	0.57±0.03**a**	0.53±0.60**a**	0.55±0.58**b**
**Fruit Firmness (g/mm)**				
2019	324.11±43.3**a**	678.41±57.5**a**	607.21±55.6**a**	630.87±36.6**a**
2020	332.15±109.6**a**	529.46 ±41.7**b**	388.39 ±53.1**b**	303.09±21.3**b**
**Juice Brix (%)**				
2019	12.95±0.30**a**	12.03±0.05**a**	12.35±0.40**a**	11.48±0.49**a**
2020	11.98±0.35**b**	12.25±1.52**a**	12.35±0.40**a**	10.58±1.00**a**
**Juice acidity (%)**				
2019	1.77±0.11**a**	1.69±0.11**a**	1.68±0.07**a**	1.81±0.161**a**
2020	1.45±0.06**b**	1.52±0.13**a**	1.68±0.06**a**	1.36±0.20**b**
**TSS/acid Ratio**				
2019	7.32±0.52**b**	7.13±0.43**a**	7.34±0.18**a**	6.39±0.75**b**
2020	8.29±0.35**a**	8.07±0.91**a**	7.35±0.14**a**	7.89±1.41**a**
**Juice pH**				
2019	3.33±0.05**b**	3.26±0.03**a**	3.57±0.28**a**	3.34±0.07**b**
2020	3.44±0.04**a**	3.32±0.05**a**	3.45±0.11**a**	3.56±0.10**a**

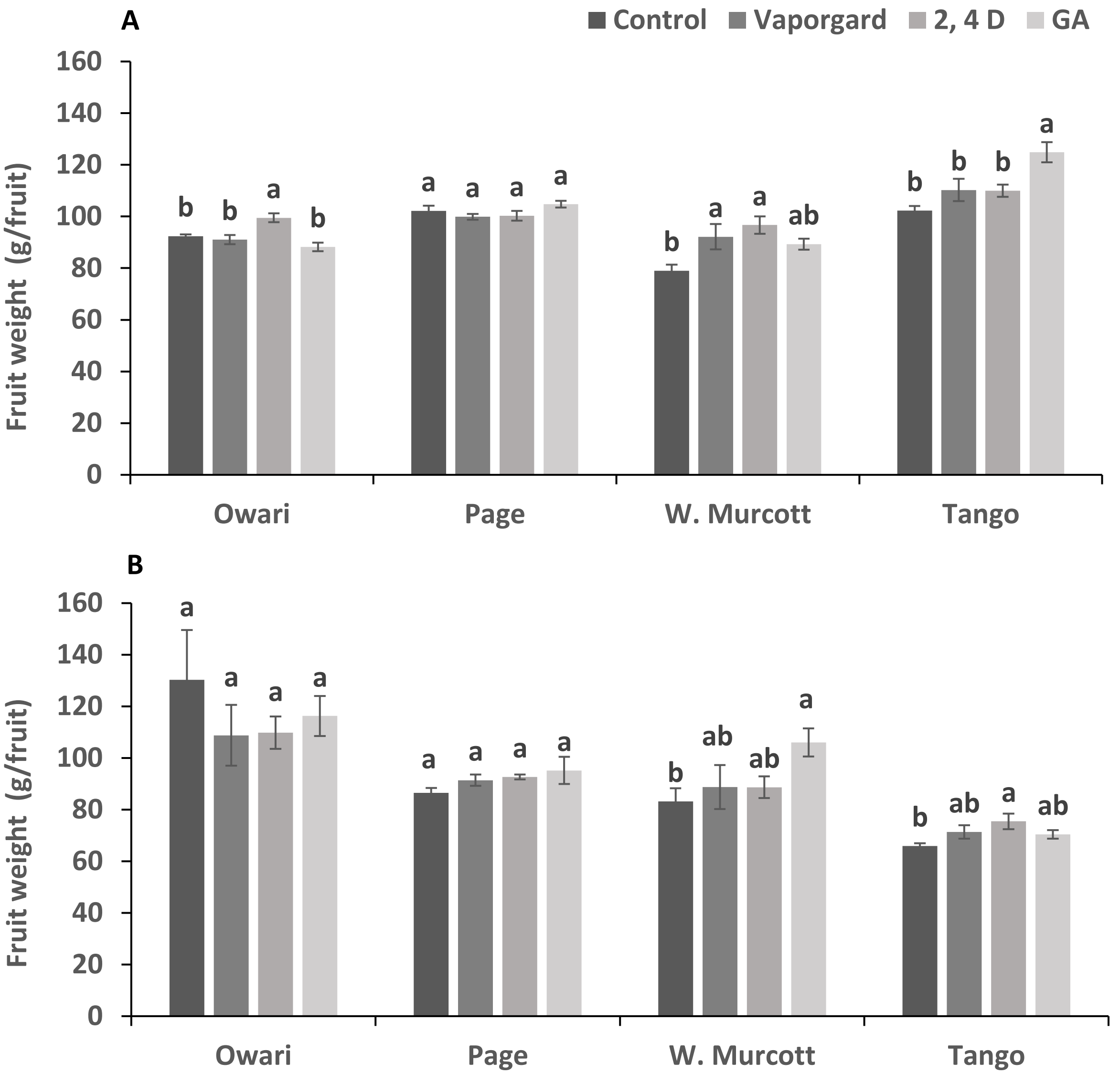

Figure S-1: Effect of various treatments on fruit weight of four mandarin varieties at harvest during the 2019 (A) and 2020 seasons (B). Letters indicate the difference among all treatments and varieties (P ≤ 0.05)

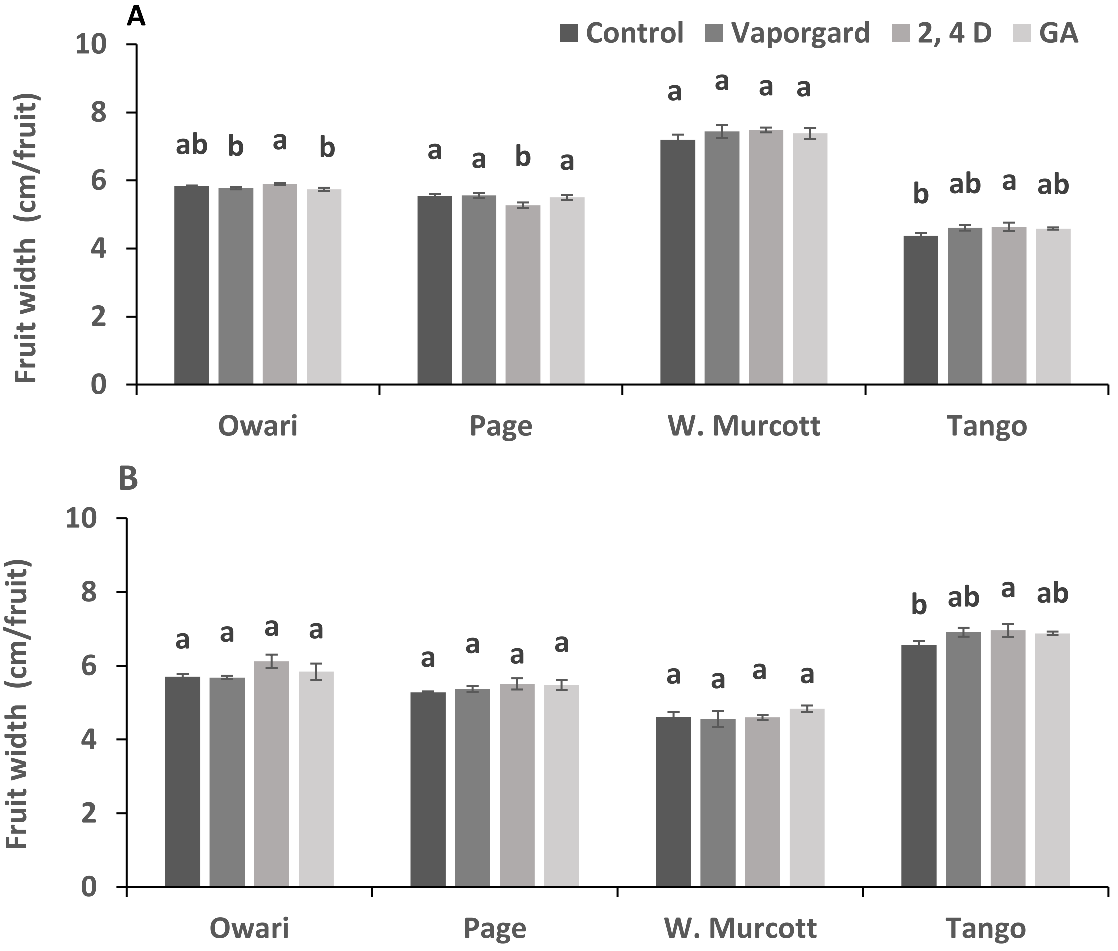

Figure S-2: Effect of various treatments on fruit width of four mandarin varieties at harvest during the 2019 (A) and 2020 seasons (B). Letters indicate the difference among all treatments and varieties ($P \leq 0.05$)

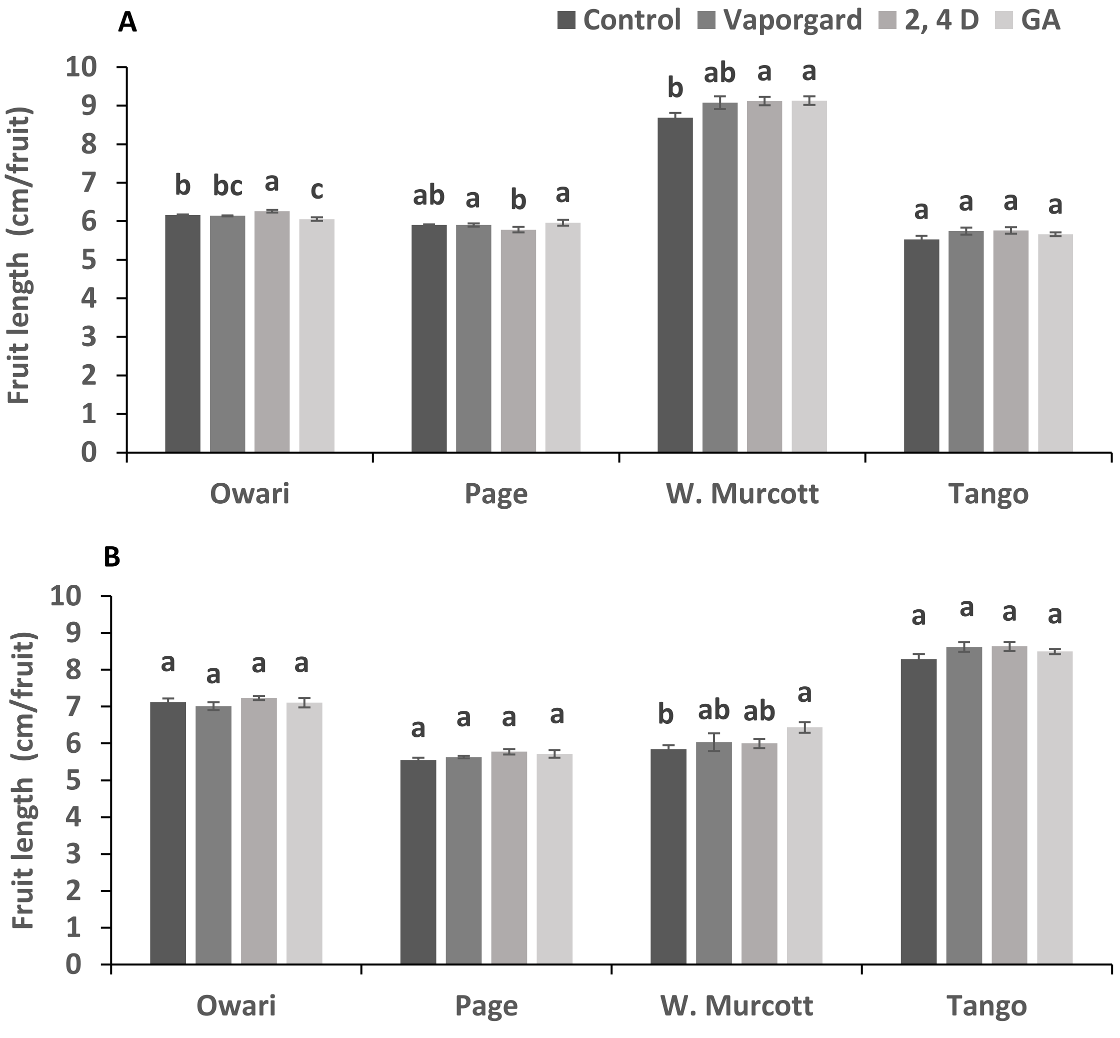

Figure S-3: Effect of various treatments on fruit length of four mandarin varieties at harvest during the 2019 (A) and 2020 seasons (B). Letters indicate the difference among all treatments and varieties (P ≤ 0.05)

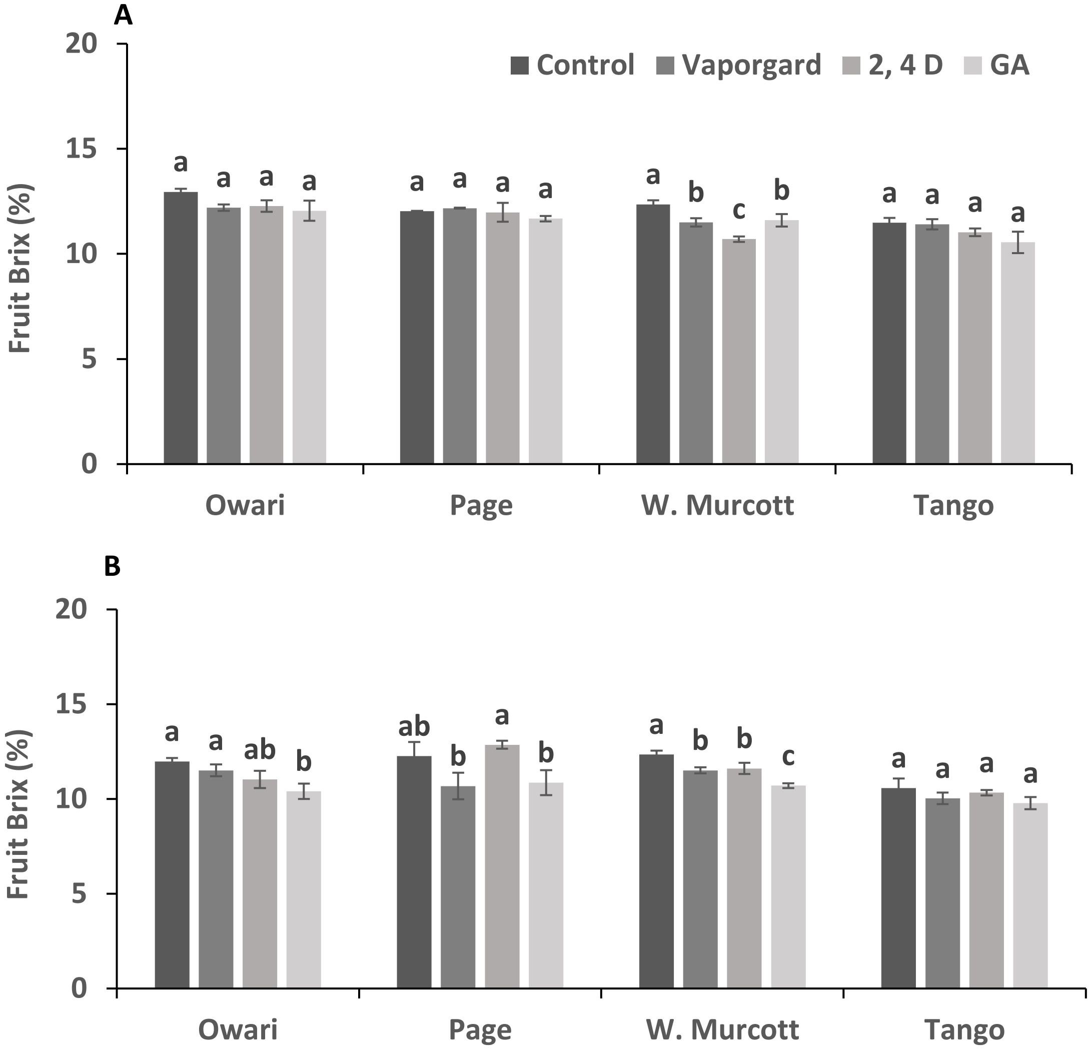

Figure S-4: Effect of various treatments on fruit sugar content (Brix) of four mandarin varieties at harvest during the 2019 (A) and 2020 seasons (B). Letters indicate the difference among all treatments and varieties ($P \leq 0.05$)

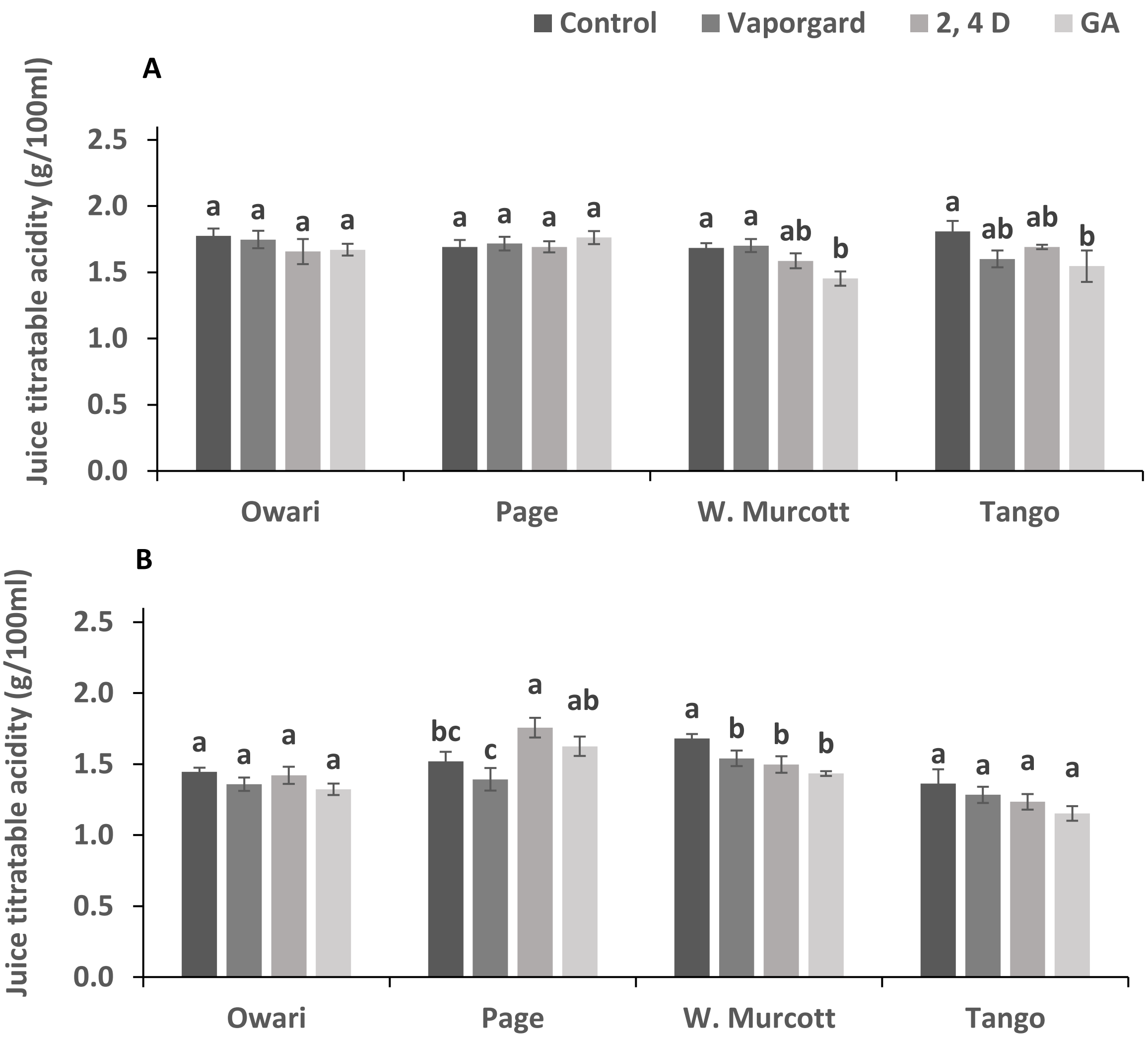

Figure S-5: Effect of various treatments on fruit titratable acidity content of four mandarin varieties at harvest during the 2019 (A) and 2020 seasons (B). Letters indicate the difference among all treatments and varieties($P \leq 0.05$)

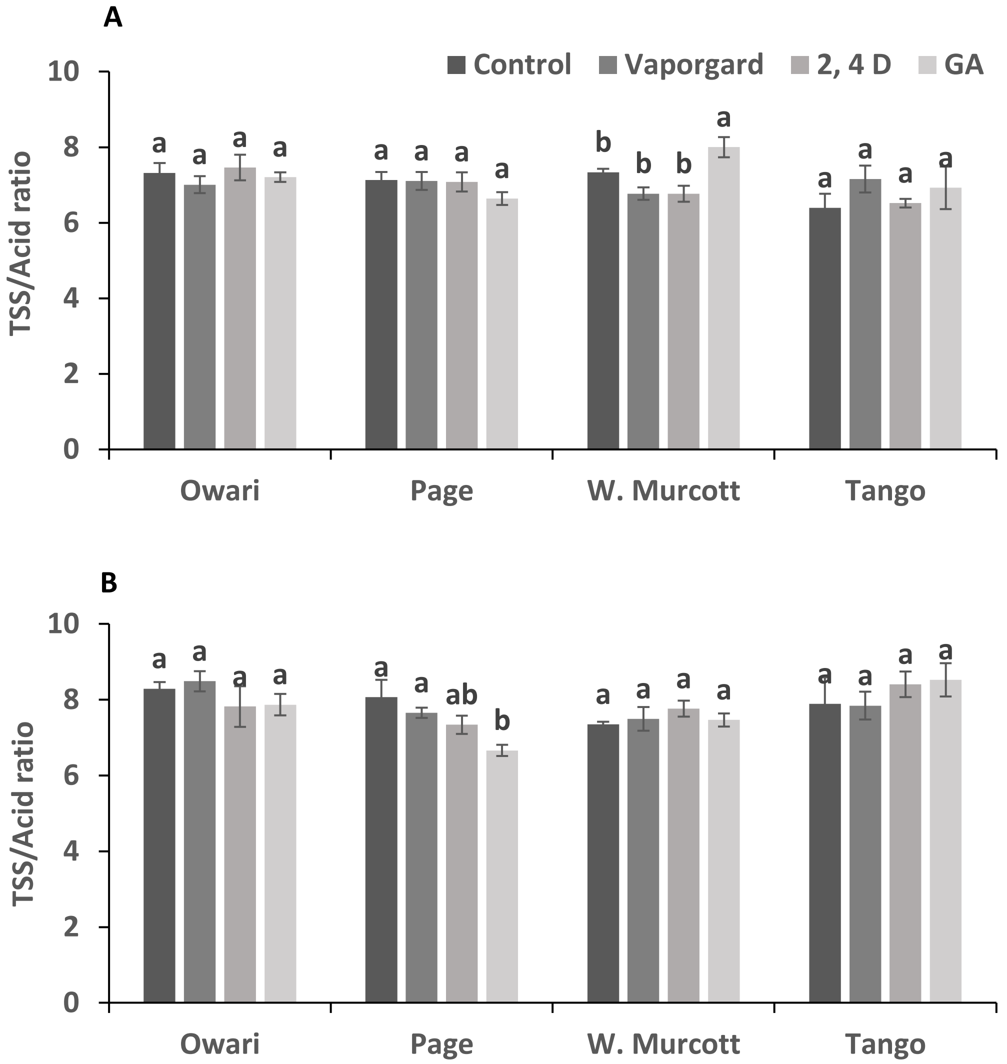

Figure S-6: Effect of various treatments on fruit TSS/Acid ratio of four mandarin varieties at harvest during the 2019 (A) and 2020 seasons (B). Letters indicate the difference among all treatments and varieties ($P \leq 0.05$)

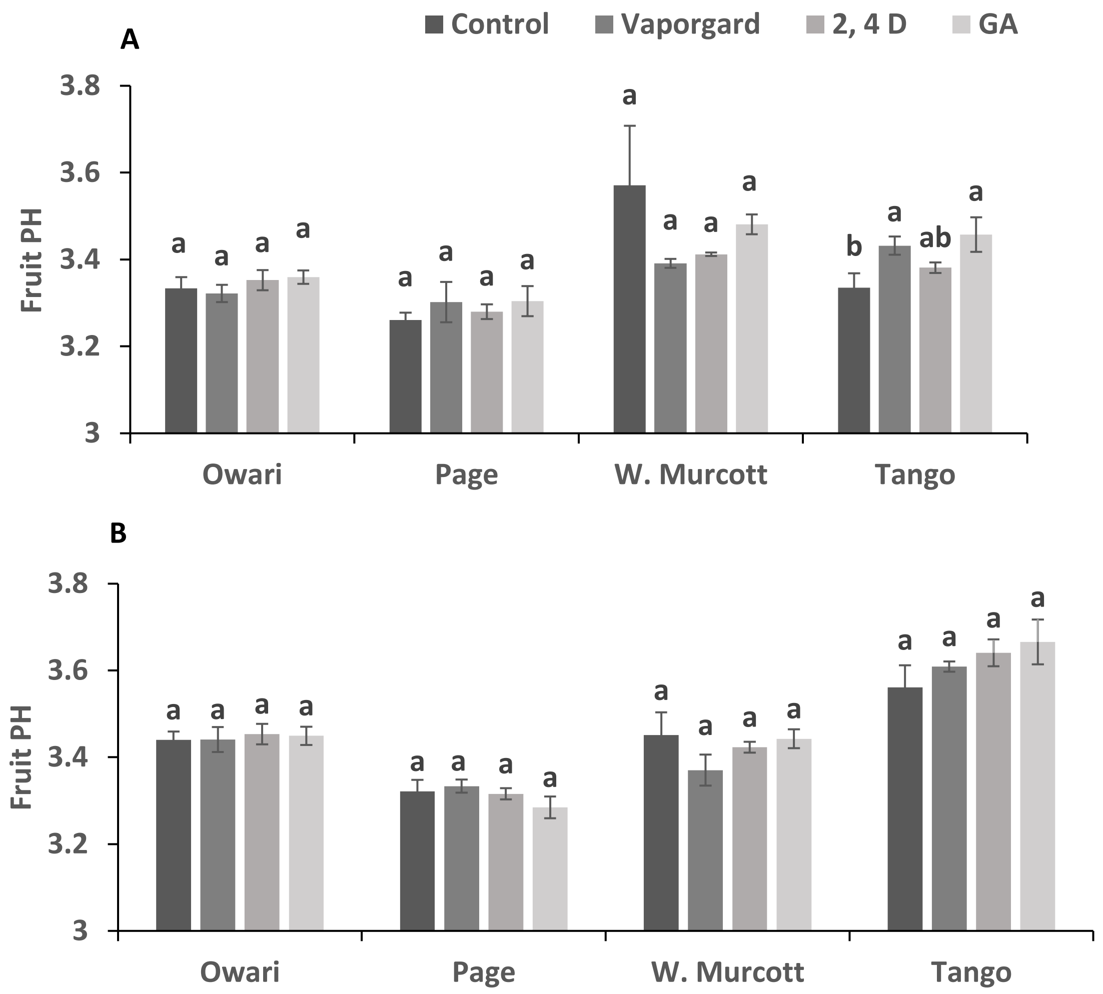

Figure S-7: Effect of various treatments on fruit PH of four mandarin varieties at harvest during the 2019 (A) and 2020 seasons (B). Letters indicate the difference among all treatments and varieties ($P \leq 0.05$)
